# Identification of key snoRNAs serves as biomarkers for hepatocellular carcinoma by bioinformatics methods

**DOI:** 10.1097/MD.0000000000030813

**Published:** 2022-09-30

**Authors:** Qingqing Xie, Di Zhang, Huifeng Ye, Zhitong Wu, Yifan Sun, Haoming Shen

**Affiliations:** a Department of Clinical Laboratory, Third Affiliated Hospital of Guangxi University of Chinese Medicine, Liuzhou, Guangxi, China; b Department of Clinical Laboratory, The Third Xiangya Hospital of Central South University, Hunan, China; c Department of Clinical Laboratory, Eighth Affiliated Hospital of Guangxi Medical University, Guigang City People’s Hospital, Guigang, Guangxi, China; d Department of Clinical Laboratory, Hunan Cancer Hospital & The Affiliated Cancer Hospital of Xiangya School of Medicine, Central South University, Hunan, China.

**Keywords:** biomarker, hepatocellular carcinoma, prognosis, risk score, snoRNA

## Abstract

Hepatocellular carcinoma (HCC) is a common malignancy with high mortality and poor prognosis due to a lack of predictive markers. However, research on small nuclear RNAs (snoRNAs) in HCC were very little. This study aimed to identify a potential diagnostic and prognostic snoRNA signature for HCC.

**Methods:**

HCC datasets from the cancer genome atlas (TCGA) and international cancer genome consortium (ICGC) cohorts were used. Differentially expressed snoRNA (DEs) were identified using the limma package. Based on the DEs, diagnostic and prognostic models were established by the least absolute shrinkage and selection operator (LASSO) regression and COX analysis, and Kaplan–Meier (K–M) survival analysis and receiver operating characteristic (ROC) curve analysis were conducted to evaluate the efficiency of signatures. Gene set enrichment analysis (GSEA) and gene set variation analysis (GSVA) were used to analyze the risk score and further explore the potential correlation between the risk groups and tumor immune status in TCGA. Gene ontology (GO) and Kyoto Encyclopedia of Genes and Genomes (KEGG) analyses were performed to determine the functions of key snoRNAs.

**Results:**

We constructed a 6-snoRNAs signature which could classify patients into high- or low-risk groups and found that patients in the high-risk group had a worse prognosis than those in the low-risk group and were significantly involved in p53 processes. Tumor immune status analysis revealed that CTLA4 and PDCD1 (PD1) were highly expressed in the high-risk group, which responded to PD1 inhibitor therapy. Additionally, a 25-snoRNAs diagnostic signature was constructed with an area under the curve (AUC) of 0.933 for distinguishing HCCs from normal controls. Finally, 3 key snoRNAs (SNORA11, SNORD124, and SNORD46) were identified with both diagnostic and prognostic efficacy, some of which were closely related to the spliceosome and Notch signaling pathways.

**Conclusions:**

Our study identified 6 snoRNAs that may serve as novel prognostic models and 3 key snoRNAs with both diagnostic and prognostic efficacy for HCC.

## 1. Introduction

Hepatocellular carcinoma (HCC) is a common cancer with a high rate of recurrence and mortality and is the fourth leading cause of cancer-related mortality worldwide.^[1,2]^ The etiology of HCC is usually related to factors that cause inflammatory liver disease, such as the hepatitis B virus, hepatitis C virus, alcohol, and food toxins.^[3]^ Clinical data show that due to the lack of accurate early diagnostic indicators, most patients at initial consultation are diagnosed at advanced stages. Due to inaccurate diagnosis and lack of effective treatment, the prognosis of most patients with HCC is poor. Therefore, it is imperative to identify new candidate biomarkers for the diagnosis, prognosis, and treatment of HCC.

Many studies have demonstrated that non-coding RNA, especially microRNAs and long non-coding RNAs, are promising biomarkers for the diagnosis and prognosis of diseases.^[4–6]^ However, small nuclear RNAs (snoRNAs) have rarely been reported as biomarkers of cancer because of their single function in the nucleolus. snoRNAs mainly exist in the nucleus, with a length of 60 to 300 NT. The main biological processes involved are rRNA processing, RNA splicing and translation regulation, and the oxidative stress response. Previous studies have shown that snoRNAs are involved in the development of various tumors.^[7]^ It has been reported that SNORD76 can promote the proliferation of tumor cells in HCC.^[8]^ Other studies have shown that SNHG3, SNHG20, SNHG6, SNORD76, and SNORA47 regulate the development of HCC cells by regulating epithelial-mesenchymal transition (EMT). In addition, SNHG16, SNORD76, and SnoU2_19 regulate HCC progression through the Wnt/β-catenin signaling pathway.^[9]^ Recently, Ding et al indicated that SNORD31, SNORA71A, and RNU5E-1 could be used as prognostic genes for HCC.^[10–12]^ However, research on snoRNA as a prognostic diagnostic marker for HCC is not comprehensive. Therefore, this study intends to use the transcriptome data of HCC from cancer genome atlas (TCGA) and international cancer genome consortium (ICGC) public databases to screen the key snoRNAs for HCC diagnosis and prognosis using bioinformatics technology.

## 2. Materials and Methods

### 2.1. Data acquisition and preprocessing

A cohort containing a total of 412 samples, including 50 normal and 362 HCC samples with gene transcriptome data and related clinical information, was extracted from TCGA database (https://portal.gdc.cancer.gov/). In total, 440 samples, including 197 normal and 243 HCC samples with complete survival information, were downloaded from the ICGC database (https://dcc.icgc.org/). snoRNAs were annotated using the Ensembl database (http://ensemblgenomes.org/). Simultaneously, snoRNAs that were differentially expressed in HCC in TCGA and ICGC datasets were screened. Then, in TCGA, the least absolute shrinkage and selection operator (LASSO) Cox regression algorithm was used to construct a diagnostic model based on differentially expressed snoRNAs (DEs). DEs with prognostic value screened by univariate Cox regression analysis were applied to construct a prognostic model with a *P* value <.05. The effectiveness of the models was evaluated by receiver operating characteristic (ROC) curve or Kaplan–Meier (K–M) survival analysis, and the prognostic model was validated using HCC samples in the ICGC database. We performed gene set enrichment analysis (GSEA) and gene set variation analysis (GSVA), emerging immunotherapy targets, and infiltrating immune cell correlation analysis among the high- and low-risk groups. Finally, we took the intersection of the diagnostic model and the prognostic model snoRNAs, plotted the single-gene ROC curve of the intersection snoRNA, and analyzed the area under the curve (AUC) > 0.6 as the key snoRNA. Finally, mRNA differentially expressed in tumor tissues were screened in TCGA, and differential genes related to key snoRNAs were identified by Pearson correlation. These genes were subjected to Gene Ontology (GO) and Kyoto Encyclopedia of Genes and Genomes (KEGG) enrichment analyses. The detailed workflow is shown in Figure S1, Supplemental Digital Content, http://links.lww.com/MD/H417.

### 2.2. Establishment and validation of the prognostic model

Survival-related snoRNAs were identified in patients with HCC, accompanied by the snoRNA expression profile as well as their clinicopathological features. Next, the intersection snoRNAs from TCGA and ICGC were screened as candidate snoRNAs that were passed on for Cox regression analysis. To further evaluate whether these candidate snoRNAs correlated with survival, 328 HCC cases with complete clinical information in the TCGA dataset were randomly divided into a training set (n = 230) and a testing set (n = 98) at a ratio of 7:3, and 243 HCC patients with complete survival information in the ICGC dataset were used as validation sets. Using univariate and multivariate Cox regression analyses, we established a prognostic signature and validated it in the training, testing, and validation sets. An snoRNA-based risk score model was constructed from this step. The risk score for each patient was calculated using the following formula.


$$\begin{array}{l} Riskscore=h0\left(t\right)*exp\left(\beta 1X1+\beta 2\;X2+\ldots +\beta nXn\right) \end{array}$$

where n is the number of predicted snoRNAs, β is the regression coefficient, and the inverse natural logarithm exp(β) gives the hazard ratio (HR). SnoRNAs with HR < 0 were considered protective factors, whereas those with HR > 0 were considered risk factors.

The patients were stratified into high- or high-risk groups according to the median risk score. We then evaluated the predictive ability of the signature for OS through K–M survival analysis as well as time-dependent ROC curve analysis conducted using the "survminer” and “survivalROC” R packages. All these processes were performed using R software (version 3.5.1).

### 2.3. Construction and evaluation of the predictive nomogram

The “rms,” nomogramEx’ nomogramEx “regplot” R package were used to construct nomogram. We constructed a nomogram to predict the survival probabilities at 1-, 2-, 3-, 4-, and 5-year survival probabilities in the TCGA cohort by integrating factors with *P* value <.05 in univariate COX analysis. Moreover, a calibration plot was used to evaluate the consistency between the predicted survival probability and the real observations.

### 2.4. GSEA and GSVA

GSEA is an approach to identify specific pathways or processes that are overrepresented in predefined subgroups, which is an alternative to differentially expressed genes (DEG)-based functional analysis.^[13]^ To explore the enrichment of KEGG pathway in the high-risk and low-risk groups, we performed GSEA analysis on 328 HCC samples in TCGA under NOM *P* value <.05. GSVA, known as GSVA, is a nonparametric unsupervised analysis method used to evaluate gene set enrichment in microarrays and transcriptome.^[14]^ GSVA was used to further analyze the difference of pathways between the subtypes using GSVA “limma” package. Differential analysis of GSVA scores in the high- and low-expression groups was performed with the low-expression group as the reference group and the difference filter condition for |*t* value|>2, *P* value <.05. When *t* > 0, we reasoned that this pathway was activated in the high expression group and vice versa. *t* < 0, we believe that this pathway is activated in the low-expression group.

### 2.5. Immune-related analysis

Using the “GSVA” R package, we calculated the enrichment score of 24 immune-related cells as well as 24 immune-related functions for each patient using single-sample gene set enrichment analysis (ssGSEA). The enrichment score was calculated in the gene expression matrix using the ssGSEA algorithm and the enrichment scores were normalized for subsequent analyses. The enrichment scores for diverse immune cells and functions of patients in different risk groups were then compared to illustrate the potential immune infiltration status. A Spearman correlation analysis heat map of the immune cells and 6 prognostic snoRNAs in HCC was generated. Correlations were considered significant and positive when *P* < .05 and *R* > 0.20. Furthermore, the expression of more than 18 immune checkpoint genes was analyzed in the different risk groups. The tumor immune dysfunction and exclusion (TIDE) framework was used to analyze the immunotherapy response of 328 HCC samples in TCGA. To identify markers with predictive value in immunotherapy response, the SubMap algorithm was used to analyze the response of high- and low-risk groups to immune checkpoint inhibitors (CTLA4 and PD1).

### 2.6. Construction of the diagnostic model

We used R package “glmnet” to perform LASSO regression analysis on DEs in HCC and normal control samples of TCGA, and constructed the diagnostic model using the selected features snoRNAs. ROC curves were used to analyze the predictive efficacy of the diagnostic model, and the AUC was calculated.

### 2.7. Identification of the key snoRNAs

First, we drew a Venn diagram of the snoRNAs that constructed the diagnostic and prognostic models to explore the intersection of snoRNAs. Then, we plotted the single ROC curve of the intersection snoRNA in the TCGA and ICGC datasets and snoRNAs with AUC > 0.6 were identified as the key snoRNAs.

### 2.8. Enrichment analysis of DEG related to key snoRNAs

To explore the DEG related to HCC, DEG analysis was performed using the edgeR package under the condition |log2FC| >1 and *P* < .05. Pearson correlation was conducted between the DEGs and the key snoRNAs, and the top 20 significant DEGs ranked by adjusted *P* values were selected. GO biological process and KEGG pathway analyses were applied to identify the potential functions of these DEGs using the clusterProfiler R package.^[15]^

## 3. Results

### 3.1. Identification of differentially expressed snoRNAs in HCC

**Figure 1.**
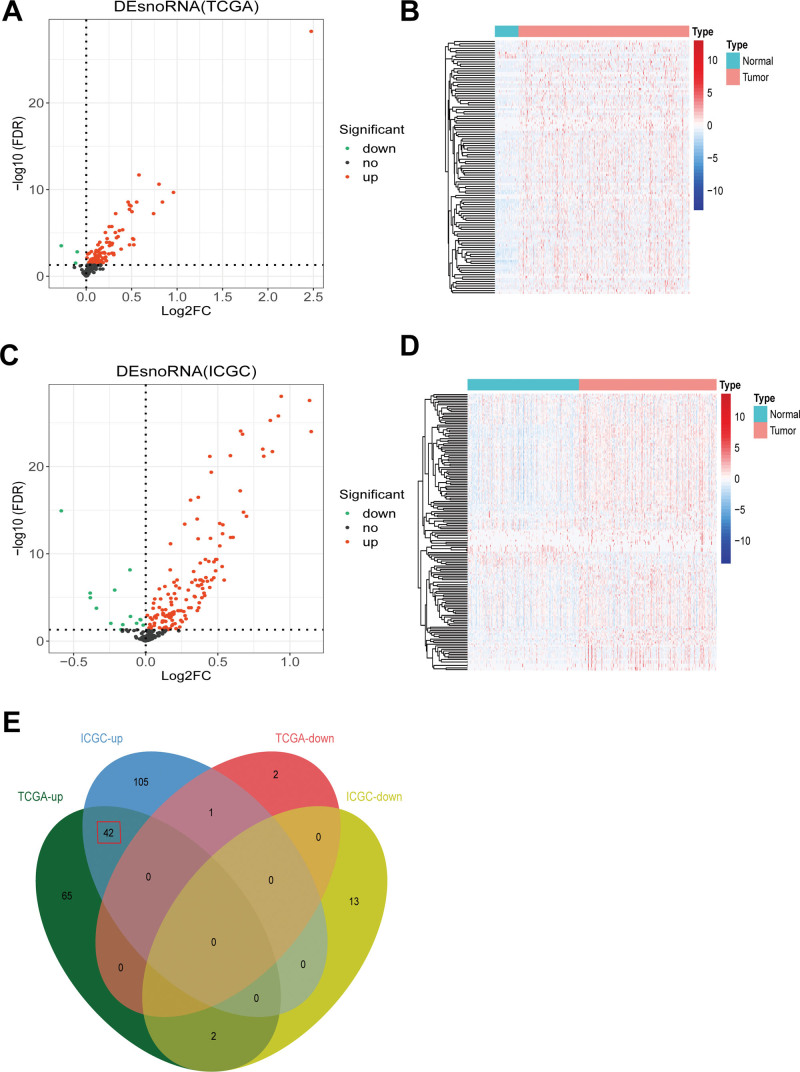
Identification of differentially expressed snoRNA in HCC. (A) Volcano plot showed the number of differentially expressed snoRNAs in TCGA. (B) Heatmap of differentially expressed snoRNAs in TCGA datasets. (C) Volcano plot showed the number of differentially expressed snoRNAs in ICGC. (D) Heatmap of differentially expressed snoRNAs in ICGC datasets. (E) An intersection analysis of differentially expressed snoRNAs in TCGA and ICGC was conducted. HCC = hepatocellular carcinoma, ICGC = international cancer genome consortium, snoRNAs = small nuclear RNAs, TCGA = the cancer genome atlas.

We identified 112 DEs, including 109 upregulated and 3 downregulated snoRNAs, in the TCGA dataset. The differential expression map of TCGA is shown in Figure 1A and the thermogram is shown in Figure 1B. We also identified 163 DEs in the ICGC dataset, including 148 upregulated and 15 downregulated snoRNAs (Fig. 1C and D). Finally, we analyzed the intersection of TCGA and ICGC DEs and obtained 45 DEs. The expression trends of the 3 snoRNAs in TCGA and ICGC were inconsistent; therefore, they were abandoned. Therefore, we used the remaining 42 snoRNAs to conduct a follow-up analysis, and all of them were found to be upregulated, as shown in Figure 1E.

### 3.2. Construction and validation of the prognostic model of snoRNA signature for HCC

**Figure 2.**
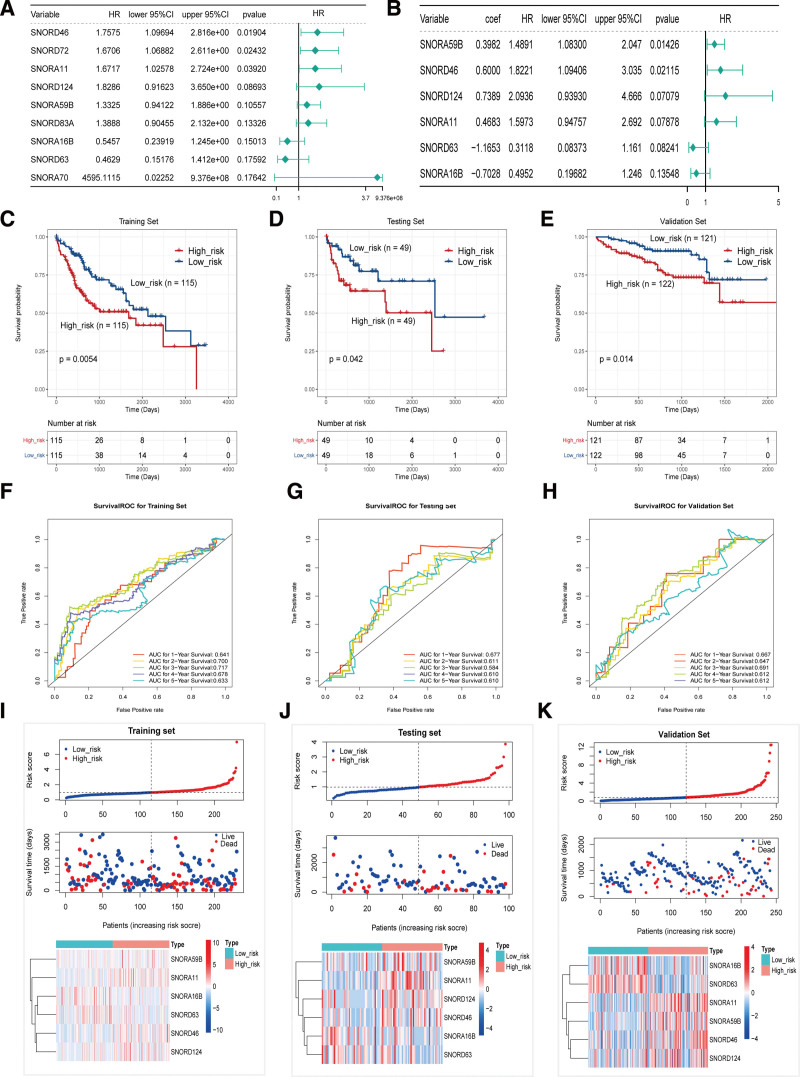
Construction and validation of the prognostic model of snoRNA signature for HCC. (A) The top 9 (9/42) significant survival-related snoRNAs using univariate cox analysis. (B) Six significant snoRNAs in multivariate cox analysis were screened out (*P* < .2) as candidates for the risk model construction. The Kaplan–Meier plot of the overall survival (OS) for high-risk and low-risk patients in the training data set (C), testing set (D), and validation set (E). Receiver operating characteristic analysis for the 6-snoRNA signature in predicting the patients of 1, 2, 3, 4, and 5 years OS in the training set (F), testing set (G), and validation set (H). The distribution of the riskscore, patients’ survival status as well as snoRNA expression signature in the training set (I), testing set (J), and validation set (K). A shorter survival time, more dead events and the expression value of 6 snoRNAs ascended or decreased with the elevation of the riskscore. AUC, areas under the ROC curve, HCC = hepatocellular carcinoma, snoRNAs = small nuclear RNAs.

Univariate Cox analyses showed the top 9 significant survival-related snoRNAs (SNORD46, SNORD72, SNORA11, SNORD124, SNORA59B, SNORD83A, SNORA16B, SNORD63, and SNORA70) (Fig. 2A), using *P* < .2 as the cutoff. These 9 snoRNAs were then subjected to multivariate Cox proportional hazards regression analysis, and snoRNAs with *P* < .2 were used for the risk model construction. A prognostic model based on 6 snoRNAs (SNORA59B, SNORD46, SNORD124, SNORA11, SNORD63, and SNORA16B) (Fig. 2B) was obtained, and a risk score formula was established according to their expression levels and coefficients. The 6-snoRNA risk score of each patient was calculated, and the patients were stratified into high-and low-risk groups according to the median risk score.

To reveal the potential prognostic value of the 6-snoRNA signature, K–M survival analysis was performed on the training, test, and validation sets. The results showed that the high-risk group patients had a significantly poorer prognosis than the low-risk group patients in the 3 cohorts (Fig. 2C–E). Furthermore, to confirm the validity of the obtained model, ROC analysis was conducted to evaluate the accuracy of this signature in predicting 1-, 2-, 3-, 4-, and 5-year survival. The results are shown in Figures 2F–H. Except for the AUC area of the test set in 3 years being 0.584, the AUC area of the rest was greater than 0.6 and reaches 0.7 in some years, indicating that the 6 snoRNA risk models had good efficiency. To further evaluate the predictive performance of the snoRNA signature, the distribution of the risk score, patient survival status, and snoRNA expression signature in the training, test, and validation sets was determined (Fig. 2I–K). The results showed that SNORA16B and SNORD63 were downregulated in the high-risk group, indicating that they act as protective factors, and SNORA59B, SNORA11, SNORD46, and SNORD124 were upregulated in the high-risk group, indicating that they were all risk factors for HCC. In conclusion, this 6-snoRNA signature can distinguish high-risk patients from low-risk patients with HCC, indicating its prognostic significance in HCC.

### 3.3. Prognostic value of the 6-snoRNA signature is independent of conventional clinical factors

**Figure 3.**
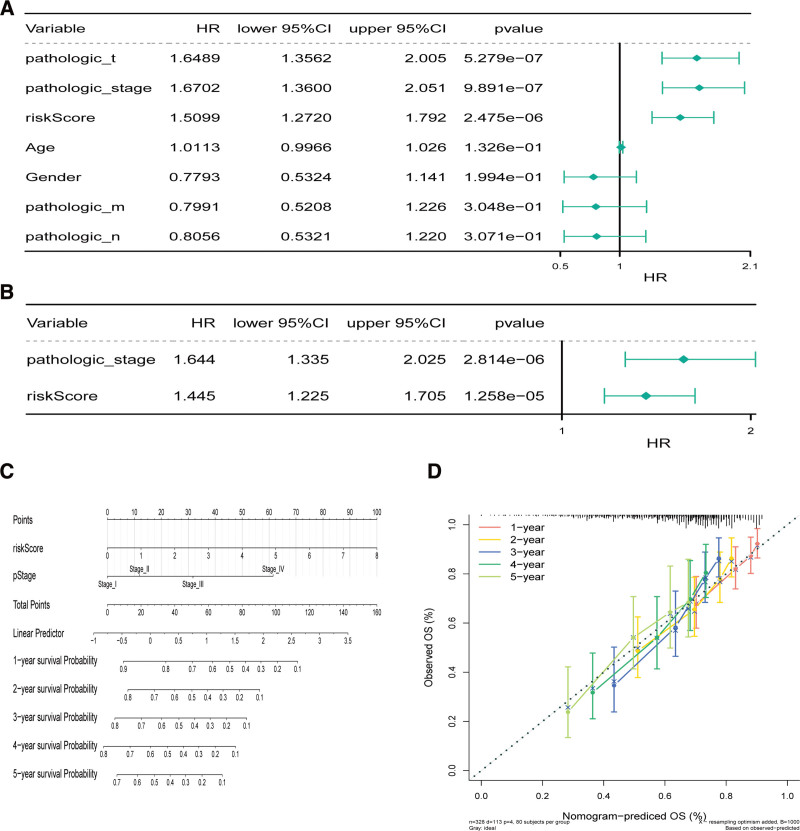
Prognostic value of the 6-snoRNAs signature is independent of conventional clinical factors. The riskscore and clinicopathological characteristics using (A) univariate cox analysis, (B) multivariate cox regression analyses. (C) Nomograms combining 6-snoRNAs signature and pathological features to predict 1-, 2-, 3-, 4-, and 5-years survival probability of patients with HCC. (D) Correction of the characteristic curve based on riskscore and pathological characteristic. HCC = hepatocellular carcinoma, snoRNAs = small nuclear RNAs.

We further appraised the predictive effect of the 6-snoRNA signature and other clinicopathological characteristics on survival status. We performed univariate and multivariate Cox analyses to determine whether the 6-snoRNA signature could be an independent risk factor for evaluating the prognosis of HCC patients. The results showed that pathological stage, T stage, and risk score were significantly related to patient survival status (all *P* < .05) (Fig. 3A). Multivariate Cox regression analysis showed that the 6-snoRNA signature and pathological stage were independent prognostic factors (Fig. 3B).

We then used pathological stage and risk score to construct a nomogram (Fig. 3C) and the 1 to 5-year correction curves (Fig. 3D). The 1 to 5-year survival rate was predicted based on the total score. The higher the score, the lower the survival rate. Thus, it can be concluded that the nomogram model has a certain prediction effect.

### 3.4. GSEA enrichment and GSVA in high and low risk groups

To explore the KEGG pathway in the high-risk and low-risk groups, 328 patients with TCGA HCC in the high-risk and low-risk groups were analyzed by GSEA at *P* < .05. A total of 27 KEGG pathways were screened, of which 13 were enriched in the high-risk group (normalized enrichment score > 0) and 14 were enriched in the low-risk group (normalized enrichment score  < 0) (Table 1). Several cancer-related processes, including the P53 signaling pathway and the cell cycle, were significantly enriched in the high-risk group. Thus, these 6 snoRNAs may be associated with tumor progression.

**Table 1:** The results of GSEA (KEGG pathways).

NAME	NES	NOM *P* val	FDR *q* val
KEGG_SPLICEOSOME	1.9529938	0	0.12383109
KEGG_RNA_POLYMERASE	1.8508013	.001968504	0.2585907
KEGG_RIBOSOME	1.838765	.005836576	0.19020182
KEGG_PYRIMIDINE_METABOLISM	1.7589208	.005825243	0.3150124
KEGG_CYTOSOLIC_DNA_SENSING_PATHWAY	1.7063819	.023529412	0.37924144
KEGG_CELL_CYCLE	1.7022977	.015444015	0.32390228
KEGG_BLADDER_CANCER	1.6731111	.012269938	0.35460255
KEGG_PATHOGENIC_ESCHERICHIA_COLI_INFECTION	1.6725879	.01775148	0.3114592
KEGG_PURINE_METABOLISM	1.6645832	.003853565	0.2918993
KEGG_RNA_DEGRADATION	1.6367117	.017175572	0.32317168
KEGG_HOMOLOGOUS_RECOMBINATION	1.6273555	.0317757	0.31436878
KEGG_P53_SIGNALING_PATHWAY	1.6000633	.015625	0.34605318
KEGG_GLYCEROPHOSPHOLIPID_METABOLISM	1.5169243	.022916667	0.40110108
KEGG_RETINOL_METABOLISM	-2.0041592	.001953125	0.037037235
KEGG_COMPLEMENT_AND_COAGULATION_CASCADES	-1.9880582	.005964215	0.023010917
KEGG_VALINE_LEUCINE_AND_ISOLEUCINE_DEGRADATION	-1.9735168	.005847953	0.01890336
KEGG_PROPANOATE_METABOLISM	-1.9710437	.001926782	0.014520246
KEGG_BUTANOATE_METABOLISM	-1.8493804	.009940358	0.05281399
KEGG_PEROXISOME	-1.8470466	.013539651	0.044542238
KEGG_FATTY_ACID_METABOLISM	-1.839958	.015355086	0.04063339
KEGG_DRUG_METABOLISM_CYTOCHROME_P450	-1.7819082	.018108651	0.058401138
KEGG_BETA_ALANINE_METABOLISM	-1.7790644	.020992367	0.053062357
KEGG_GLYCINE_SERINE_AND_THREONINE_METABOLISM	-1.7447815	.029296875	0.06632929
KEGG_ASCORBATE_AND_ALDARATE_METABOLISM	-1.7309594	.016194332	0.06751746
KEGG_PRIMARY_BILE_ACID_BIOSYNTHESIS	-1.7269616	.01417004	0.06440593
KEGG_TRYPTOPHAN_METABOLISM	-1.6852603	.03875969	0.08126818
KEGG_STARCH_AND_SUCROSE_METABOLISM	-1.5867703	.03629032	0.13525608

GSEA = gene set enrichment analysis, KEGG = Kyoto encyclopedia of genes and genomes, NES = normalized enrichment score.

**Figure 4.**
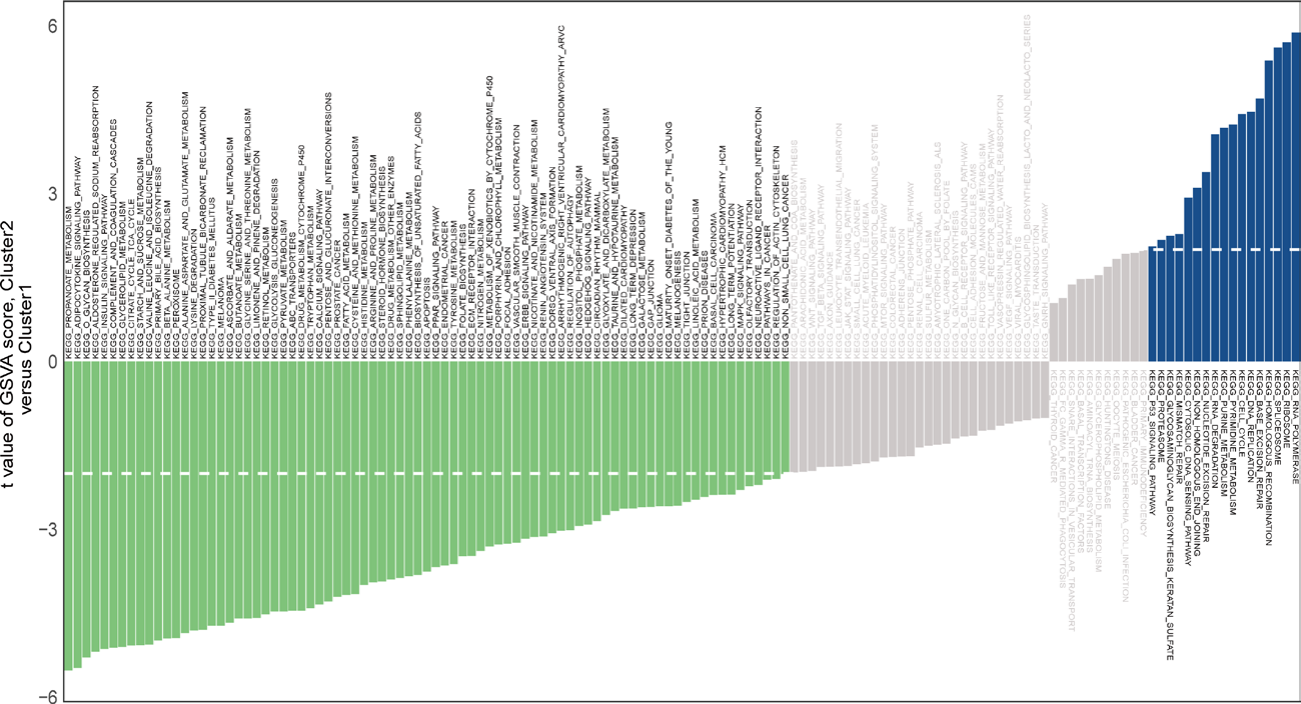
The GSVA analysis based on the KEGG pathway. The *t* values of each pathway in the high and low risk group on the vertical axis to represent the difference values. Negative values (green) indicate activation of the pathway in the low expression group, and positive values (blue) indicate activation of the pathway in the high expression group. GSVA = gene set variation analysis, KEGG = Kyoto encyclopedia of genes and genomes.

To explore the KEGG pathway activated in the high-risk and low-risk groups, we performed GSVA analysis on 328 high-risk group samples of TCGA HCC using the KEGG pathway as the preset pathway (Fig. 4). Finally, we identified 17 pathways that were activated in the high-risk group and 81 pathways that were activated in the low-risk group. It is interesting to note that the P53 pathway was activated in the high-risk group, which is consistent with the GSEA results.

### 3.5. Analysis of immune cell infiltration, immune checkpoints and the response to immunotherapy

**Figure 5.**
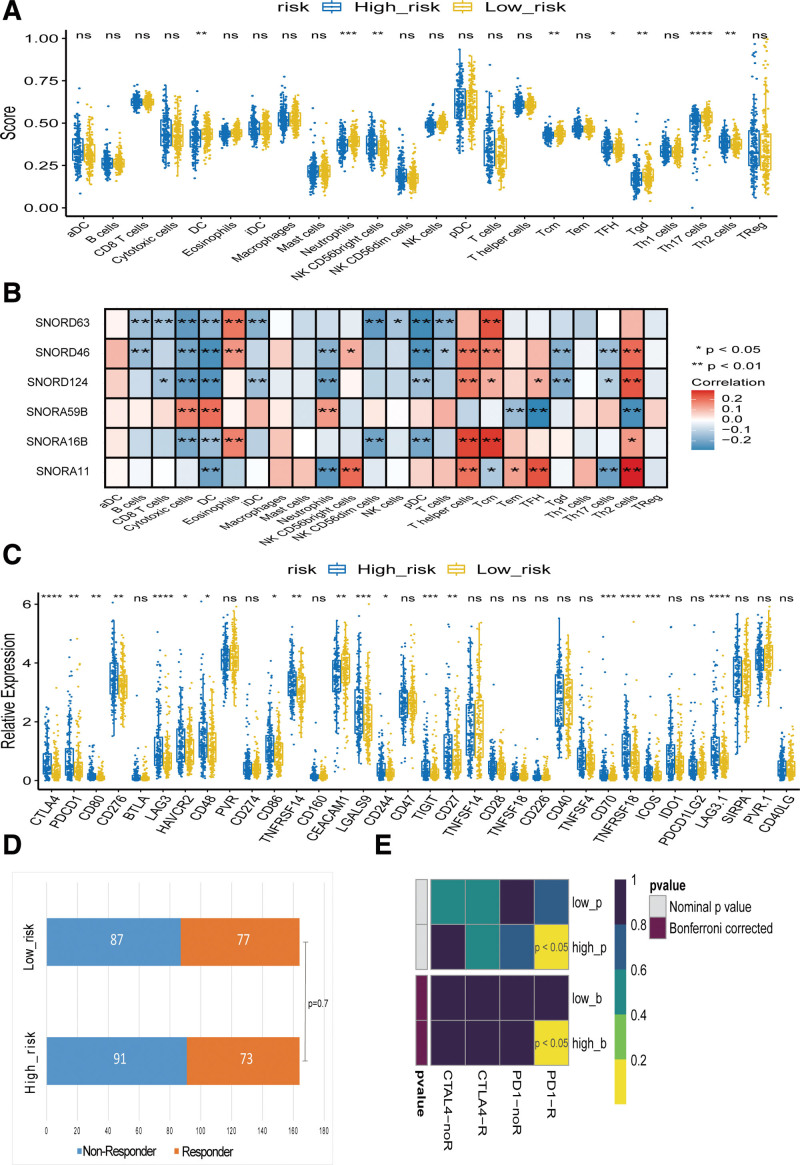
Analysis of immune cell infiltration, immune checkpoints and the response to immunotherapy. (A) The risk score significantly correlated with the infiltration levels of various immune cells. (B) The expression 6 snoRNAs significantly correlated with the infiltration levels of various immune cells. (C) Correlation analyses of the risk groups with immune checkpoint genes (D) Analysis of responses to TIDE immunotherapy. (E) Analysis of responses to immunosuppressive agents. *P < .05, **P < .01, ***P < .001, and ****P < .0001. snoRNAs = small nuclear RNAs, TIDE = tumor immune dysfunction and exclusion.

Although GSEA and GSVA analyses were not enriched in immune-related pathways, we still attempted to explore whether the risk model based on DEs was related to immunity. We conducted ssGSEA immune cell infiltration analysis, immune checkpoint analysis, and TIDE immunotherapy response analysis on 328 samples of TCGA HCC. Simultaneously, the SubMap algorithm was used to analyze the response of high- and low-risk groups to immune checkpoint inhibitors (CTLA4 and PD1). As shown in Figure 5A, the risk score is significantly associated with the abundance of infiltrating immune cells. The NK CD56bright cells, TFH, Th17 cells, and Th2 cells were more infiltrated in the high-risk group, while DC, neutrophils, and Tcm infiltrated more in the low-risk group.

We further analyzed the correlation between the 6 snoRNAs and immune cells in the prognostic model. Consistently, the expression of all 6 snoRNAs was associated with DC infiltration in HCC. The expression of SNORD46, SNORD124, SNORA59B, and SNORA11 significantly correlated with neutrophil infiltration. The expression of SNORD46 and SNORA11 positively correlated with the infiltration of NK CD56bright cells. The expression of SNORD63, SNORD46, SNORD124, SNORA16B, and SNORA11 is related to the infiltration of Tcm. The expression of SNORD124, SNORA59B, and SNORA11 was related to the infiltration of TFH, while the expression of SNORD46 and SNORD124 was negatively related to the infiltration level of Tgd. The expression of SNORD46, SNORD124, and SNORA11 was negatively correlated with the infiltration level of Th17 cells, while the expression of SNORD46, SNORD124, SNORA59B, SNORA16B, and SNORA11 was related to Th2 cells (Fig. 5B).

We further analyzed the correlation between the risk groups and expression levels of the immunoassay checkpoints. Both CTLA4 and PDCD1 (PD1), which are the most important checkpoints, were highly expressed in the high-risk group. The results showed that 18 immunoassay checkpoints were differentially expressed in the high and low risk groups, and the most important immunoassay checkpoints: CTLA4 and PDCD1 (PD1) were highly expressed in the high-risk group (Fig. 5C).

Finally, to identify potential groups that may benefit from immunotherapy, we used the TIDE algorithm to predict the response to immunotherapy in the high- and low-risk groups. However, TIDE failed to predict response to immunotherapy in HCC (*P* = .7), as shown in Figure 5D. We then evaluated whether the potential HCC groups were significantly changed in response to CTLA4 and PD1 inhibitor treatment, and the results showed that the high-risk group was likely to respond to PD1 inhibitor treatment, as shown in Figure 5E, which was consistent with the results in Figure 5C.

### 3.6. Construction of the diagnostic snoRNAs signature for HCC and identified key snoRNAs

**Figure 6.**
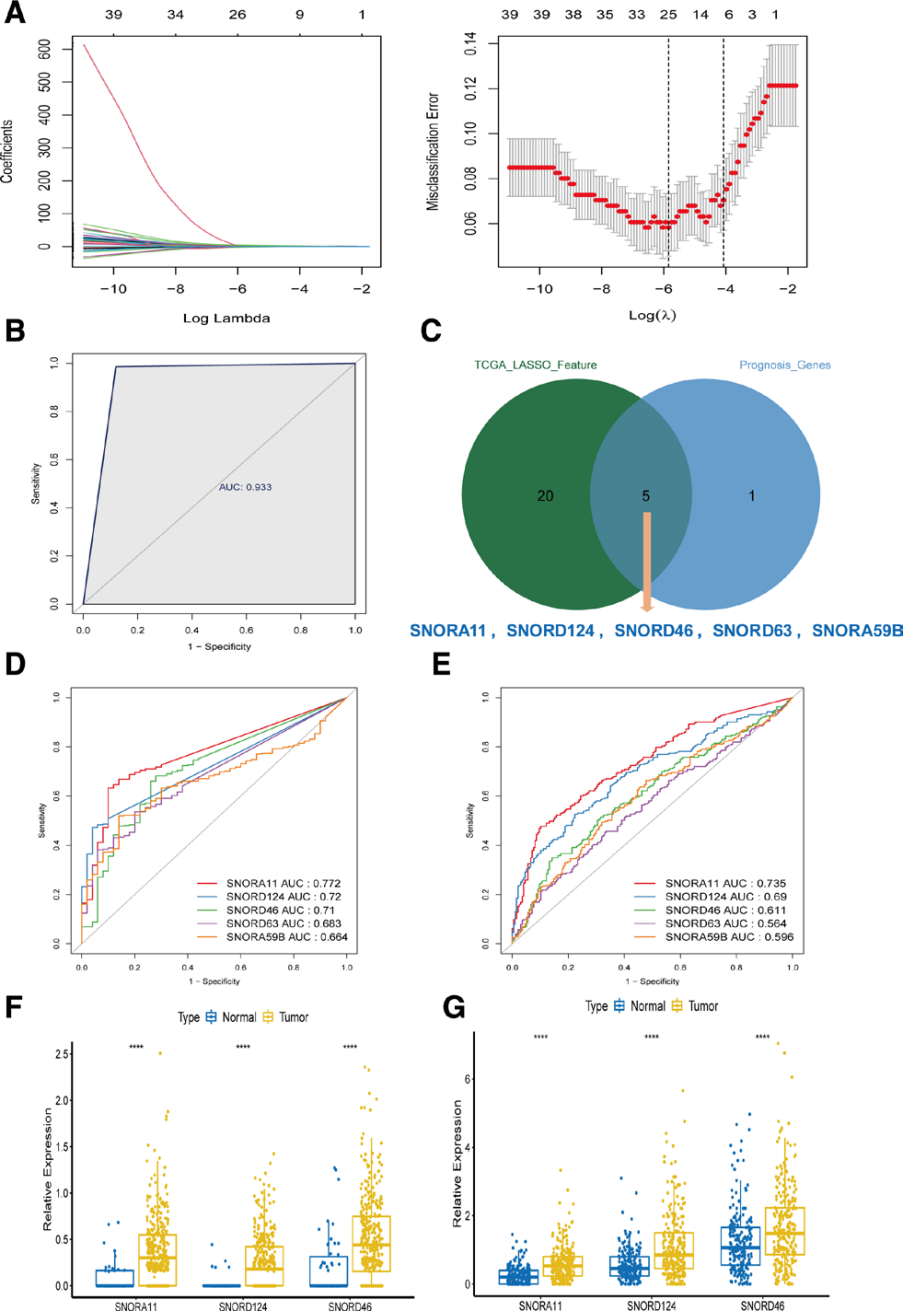
Construction of the diagnostic snoRNA signature for HCC and identified key snoRNAs (A) least absolute shrinkage and selection operator (LASSO) coefficient profiles of the 42-survival related snoRNA. The tuning parameter (lambda) selection in the LASSO model. (B) The ROC curve of LASSO regression analysis. (C) The intersection diagram of 25 diagnostic model snoRNAs and 6 prognostic model snoRNAs. ROC curves for the SNORA11, SNORD124, SNORD46, SNORD63, SNORA59B in TCGA (D), ICGC (E). Key snoRNA expression level of SNORA11, SNORD124, SNORD46 in TCGA(F), ICGC (G). ****P < .0001. HCC = hepatocellular carcinoma, ICGC = international cancer genome consortium, ROC = receiver operating characteristic, snoRNAs = small nuclear RNAs, TCGA = the cancer genome atlas.

A diagnostic model was constructed on 42 DEs using LASSO regression from TCGA, and 25 snoRNAs were selected using the minimum criteria (Fig. 6A). We plotted the ROC curve of the model and evaluated its ability to predict normal and cancerous samples (Fig. 6B). The area under the ROC curve was 0.933, indicating that the 25-snoRNAs signature-based diagnostic model could distinguish normal samples from HCC samples with HCC, indicating its diagnostic significance for HCC.

Then, we took the intersection of 25 diagnostic snoRNAs and 6 prognostic model snoRNAs, and obtained 5 snoRNAs, including SNORA11, SNORD124, SNORD46, SNORD63, and SNORA59B (Fig. 6C). We plotted the single ROC curves of 5 snoRNAs in the TCGA and ICGC datasets and chose the snoRNAs with AUC > 0.6 as the final key snoRNAs. Whether in the TCGA or the ICGC data set, the AUC for SNORA11, SNORD124, and SNORD46 were all greater than 0.6 (Fig. 6D and E). The expression levels of the 3 key snoRNAs in TCGA and ICGC datasets are shown in Figure 6F and G.

### 3.7. snoRNAs associated differentially expressed mRNAs and their functions

**Figure 7.**
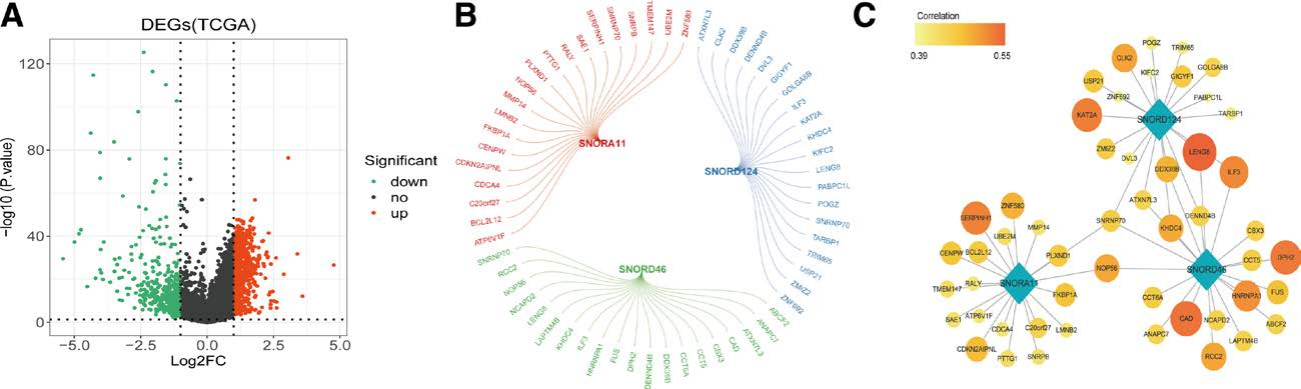
Identification of differentially expressed mRNAs relating key snoRNAs in HCC. Volcano plot of differentially expressed mRNAs in HCC. (B) Pearson correlation analysis between key snoRNA and differentially expressed mRNAs. (C) Cytoscape visualization of the snoRNA-mRNA regulatory network. The diamonds represented snoRNAs, and circles represented differential mRNAs. The correlation of red representation was relatively high, and that of yellow representation was relatively low. The size of the point also represented the correlation, the larger the point, the greater the correlation. HCC = hepatocellular carcinoma, snoRNAs = small nuclear RNAs.

To determine differential mRNA expression profiles in HCC, we screened for mRNA expression in the TCGA dataset. A total of 1367 DEG were screened, of which 973 were upregulated and 394 were downregulated. A volcano map of the DEGs is shown in Figure 7A.

We performed Pearson correlation analysis of differential genes with 3 key snoRNAs under the settings of *P* < .05, the TOP20 related differential genes were considered as the key snoRNAs related mRNAs, as shown in Figure 7B. All related mRNAs were positively correlated. Cytoscape was used to visualize the snoRNA-mRNA network with 54 nodes and 60 edges (Fig. 7C).

**Figure 8.**
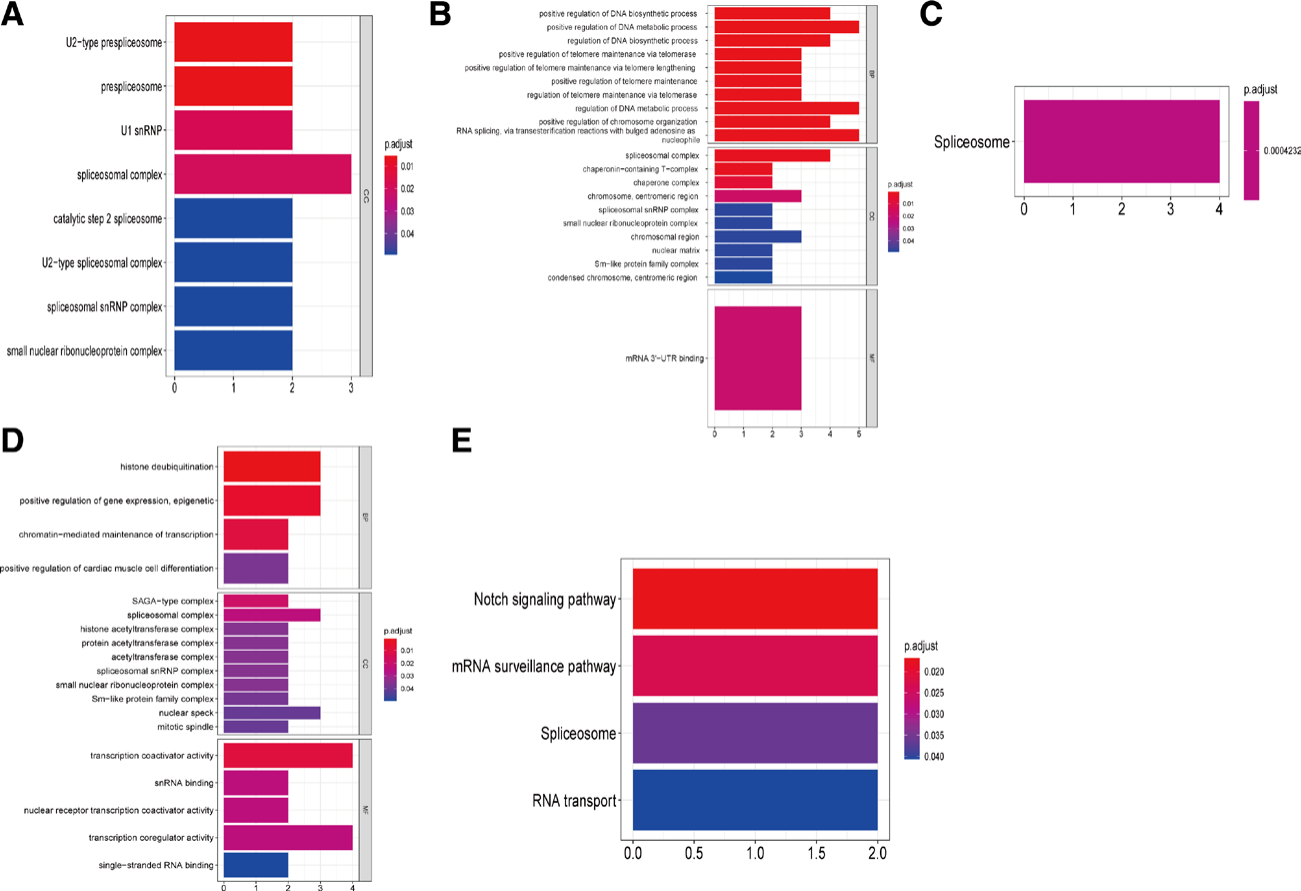
Functional enrichment analysis of key snoRNAs relating genes in HCC. GO enrichment analysis of top 20 different mRNAs related to SNORA11(A), SNORD46(B), SNORD124 (D). The horizontal axis represents the gene proportion of the GO entry, that is, the number of differential genes contained in the GO entry; The vertical axis represents the name of the GO entry and the color representation -log10P-value. KEGG enrichment analysis of 20 different mRNAs associated with SNORD46 (C), SNORD124 (E). The horizontal axis represents the proportion of genes in the KEGG pathway, that is, the number of differential genes in the pathway. The vertical axis represents the name of the KEGG pathway and the color representation -log10 *P* value. GO = gene ontology, HCC = hepatocellular carcinoma, KEGG = Kyoto encyclopedia of genes and genomes, snoRNAs = small nuclear RNAs.

To deeply examine the molecular mechanisms and related pathways of the top 20 related mRNAs, we performed GO enrichment analysis and KEGG analysis for HCC.As for SNORA11, the top 20 related proteins were enriched into 8 “CC” GO terms. However, no significant terms in the KEGG analysis were found (Fig. 8A); As for SNORD46, the GO enrichment analysis identified 70 enriched GO terms (59 BP, 1 MF, and 1 CC). We sorted by *P* value and visualized the top10 entries of the directory, as shown in Figure 8B. In addition, we enriched into 1 KEGG pathway: spliceosome, as shown in Figure 8C; As for SNORD124, the top 20 related proteins were enriched into 21 “CC,” 5 “MF,” and 4 “BP” GO terms, respectively. We visualized the top 10 of each directory in *P*-value order, as shown in Figure 8D. In addition, we enriched the following 4 KEGG pathways: Notch signaling pathway, mRNA surveillance pathway, spliceosome, and RNA transport, as shown in Figure 8E.

## 4. Discussions

The prognosis and survival of patients is still poor. Therefore, there is an urgent need for accurate early diagnosis and long-term prognostic prediction of molecular screening for HCC biomarkers. This study highlights the prognostic and diagnostic value of snoRNAs and explores their underlying functions. We identified 6 snoRNAs that were significantly related to the prognosis of HCC by data mining and data analysis and constructed a prognostic signature based on 6 prognostic snoRNA expression values (SNORA59B, SNORD46, SNORD124, SNORA11, SNORD63, and SNORA16B). GSEA and GSVE of the risk score suggested that the high-risk score phenotype was closely related to the p53 signaling pathways. We also screened 3 key snoRNAs with diagnostic and prognostic functions and found that some of them were closely related to the spliceosome and Notch signaling pathways. These results may provide new prognostic, diagnostic, and therapeutic implications for HCC patient management.

Along with an increasing number of mechanisms of HCC tumorigenesis being revealed, newly discovered biomarkers have been evaluated for the diagnosis, assessment, and treatment of HCC.^[16]^ In recent years, mounting evidence has indicated a direct relationship between ncRNA dysfunction and tumor oncogenesis, as well as the function of ncRNAs as assessment indicators for tumor progression. Among these, snoRNAs, a type of ncRNA with a length of 200 nucleotides, are commonly found in the nucleolus and perform regulatory functions in the process of post-transcriptional modification.^[17]^ Following advances in the field of tumor regulation, it has been proven that the dysfunction of specific snoRNAs can directly induce and promote the development of various tumors,^[18]^ including HCC. Xu et al were the first to illustrate the relationship between snoRNA and HCC, which indicated that snoRNA SNORD113-1 in HCC could inactivate the intracellular phosphorylation of ERK1/2 and SMAD2/3, demonstrating its tumor-suppressing function.^[19]^ In contrast, SNORD126 overexpression in HCC was shown to function as a tumor-promoting snoRNA by increasing fibroblast growth factor receptor 2 expression and activating the PI3K-AKT pathway.^[20]^ Moreover, according to a recent study by McMahon et al, HCC patients with low SNORA24 expression tend to exhibit poor long-term survival.^[21]^

In recent years, the potential of snoRNAs as biomarkers has been proven to predict the clinical prognosis of different types of cancers.^[7,22–24]^ In addition, a predictive model based on several snoRNAs has been developed for several cancers. For example, Zhao et al^[25]^ screened and constructed 6-snoRNAs diagnostic and prognostic signatures using clear cell renal cell carcinoma patients in the TCGA cohort. Xing et al^[26]^ also identified 5 prognostic snoRNA signatures by performing univariate survival analysis in TCGA head and neck squamous cell carcinoma cohort. Liu et al also identified 15 prognostic snoRNAs and identified a 4-snoRNA signature for sarcoma overall survival.^[27]^ In the current study, we performed a systematic analysis of the potential role of snoRNAs in HCC and made several important discoveries. We identified 42 DEs and established 6 survival-related snoRNAs using Cox analysis: 2 of the 6 snoRNAs, SNORA16B and SNORD63, are protective factors, and the other 4 (SNORA59B, SNORD11, SNORD46, and SNORD 124) are risk factors that may play a crucial role in tumor metastasis and progression. The signature was validated in the training, testing, and validation sets, suggesting its reliability. This finding is consistent with the results of previous studies. As for SNORD63, a previous study reported that SNORD63 was greatly upregulated in urinary sediment and SNORD96A elevated in plasma could act as a noninvasive diagnostic biomarker for clear cell renal cell carcinoma.^[28]^ Recently, Liu et al^[27]^ identified 15 snoRNAs that were significantly related to sarcoma prognosis and constructed a prognostic signature based on 4 prognostic snoRNA (U3, SNORA73B, SNORD46, and SNORA26) expression values. As for SNORA16B, SNORA59B, SNORA11and SNORD124, there have been no related reports in the cancer field. However, the expression of these 6 snoRNAs and their clinical impact as biomarkers for HCC have not been investigated in previous studies. This study is the first to report these 6 prognostic snoRNAs in HCC. In this study, we also screened the 25-snoRNAs signature for diagnosis in patients with HCC for the first time, confirming that snoRNA expression levels can be used for the diagnosis of HCC.

Clinicopathological characteristics, including age, sex, pathological stage, and TNM stage, are of great importance for HCC; however, these features do not just belong to HCC. From this perspective, we successfully established a molecular scoring system based on the expression profiles of the 6 survival-related snoRNAs and assessed the reliability of the prognostic model by conducting K–M survival analysis separately in the training, test, and validation sets. We found that this signature helped stratify low-and high-risk groups and predicted the OS of patients with HCC with high sensitivity and specificity. In addition, we found that the 6-snoRNA signature and pathological stage were independent risk factors for OS in HCC patients, like previous studies.^[26,27]^ Another contribution of our research was that we integrated clinical characteristics with the 6 survival-related snoRNA signatures to construct nomogram models that could amplify the clinical value and simplify the use of this signature in clinical practice.

To determine the functional mechanism of these 6 snoRNAs, we used GSEA and GSVA to explore the function of this 6-snoRNA prognostic signature. Notably, our present study indicated that the well-known p53 signaling pathway was activated in the high-risk group, which suggests that the 6 snoRNAs can be significantly enriched in the p53 signaling pathway. Many genes promote tumor progression by inducing EMT through the p53 pathway.^[29–31]^ Indeed, snoRNA promote tumor growth and metastasis by inducing EMT in HCC.^[32]^ However, little is known about the mechanism of snoRNAs in EMT of HCC. Therefore, the role of snoRNAs in p53 signaling may provide new clues for p53-dependent cancer treatment.

The ability of cancer cells to avoid detection and clearance by the immune system has recently become a central research topic in oncology. Numerous studies have shown a link between immune cell infiltration at the tumor site and a better response to therapy and prognosis in carcinomas. We found that NK, CD56bright cells, TFH, Th17, and Th2 cells had higher infiltration in the high-risk group, while DC, neutrophils, and Tcm had higher infiltration in the low-risk group. Immune checkpoints play an important role in tumor immune escape and the formation of the tumor microenvironment. Antibody-based drugs targeting cytotoxic T-lymphocyte-associated protein 4 (CTLA-4), programmed cell death protein 1 (PD-1), and programmed cell death protein-ligand 1 (PD-L1) have proven superior to the existing chemotherapy and radiation approaches for multiple cancer types.^[33]^ Studies have shown that snoRNA expression is highly relevant to lymphocyte function and the immune response against cancers as well as the oncogenic mechanisms that promote their progression to metastatic disease.^[34]^ Interestingly, our study suggests that the high-risk prognostic group showed high expression of PD1 and CTLA4 and was more sensitive to anti-PD1 treatment. These results are similar to those of previous studies.^[33–36]^

In recent years, the potential of snoRNAs as diagnostic biomarkers has been recognized. For example, SNORA42 was identified as a novel diagnostic and predictive biomarker and a prospective therapeutic target for CRC patients. SNORD63 was greatly upregulated in urinary sediment and SNORD96A elevated in plasma acted as a noninvasive diagnostic biomarker for clear cell renal cell carcinoma.^[28]^ In our study, we aimed to construct a signature based on snoRNA expression profiles for the classification of patients with HCC, which could be more effective in distinguishing between normal and tumor patients. In the present study, we characterized the snoRNA expression profiles in HCC and identified 42 DEs. We then screened for diagnostic snoRNAs using the LASSO Cox regression method and developed an HCC diagnostic signature composed of 25 snoRNAs. Moreover, the diagnostic model was validated using ROC curve analysis, with a high AUC. Hence, the clinical value of the diagnostic models for HCC should attach importance to this point, and further investigation is needed.

To further determine whether these snoRNAs have diagnostic and prognostic value, we obtained 5 characteristic snoRNAs (SNORA11, SNORD124, SNORD46, SNORD63, and SNORA59B) by intersecting the diagnostic and prognostic models and obtained 3 key snoRNAs (SNORA11, SNORD124, and SNORD46) with high diagnostic and prognostic efficacy. Among these 3 snoRNAs, our analysis also identified all of them as differentially expressed in TCGA and ICGC HCC data. Finally, to further understand the biological function and explore the underlying oncogenic mechanism of the key snoRNAs, we first screened 1367 differentially expressed mRNAs in HCC samples in TCGA and further identified the top20 mRNAs related to key snoRNAs for further KEGG and GO enrichment analyses. Functional enrichment showed that the co-expressed genes of SNORD46, SNORD 124 can be significantly enriched in some well-known cancer-related pathways, such as the spliceosome and Notch signaling pathways. Some functions of RNA transport and mRNA surveillance are also discussed. These enrichment results have been reported to be closely related to HCC in previous studies. Lin et al^[37]^ identified 3 key genes in HCC and found that they were mainly enriched in signaling pathways involved in the spliceosome and cell cycle. In another study came to similar conclusions, researchers identified 977 proteins (DEP) and 243 DEG in HCC and found that the DEP-DEGs were mainly enriched in the spliceosome and various metabolic processes.^[38]^ The Notch signaling pathway has been found to be significantly associated with HCC cell migration, invasion, and apoptosis^[39]^ regulates the differentiation of macrophages into M1 to promote inflammation and antitumor activity.^[40]^ Our results are consistent with those of the above studies, indicating that these 2 signaling pathways are closely related to the occurrence and development of liver cancer, and the regulatory relationship between key snoRNAs requires further study.

This study has some limitations. First, the dataset that was used for the development of the diagnosis and prognosis models was obtained from TCGA and ICGC HCC cohorts, which may not completely include the clinical parameter information. This could have led to deviations in the results. Second, because the results of our study were based on data mining and data analysis, the drugs and functional mechanisms we screened were not verified experimentally in vivo or in vitro; therefore, comprehensive and accurate results could not be obtained. These theoretical predictions must be experimentally validated in future studies. Despite these shortcomings, the results of this study have important clinical implications. Our study is the first to report the screening of snoRNA prognostic and diagnostic markers for HCC from RNA-seq datasets, which provides a more comprehensive theoretical basis for future research on the clinical application value of snoRNA in HCC. At the same time, the snoRNA prognostic and diagnosis markers screened in this study and the constructed risk score model and diagnosis model are also expected to be applied in clinical practice in the future. Accordingly, our signature findings make it highly promising for further clinical applications.

## 5. Conclusions

In conclusion, our study identified 6 snoRNAs that may serve as novel prognostic models and 3 key snoRNAs with both diagnostic and prognostic efficacy for HCC. These results may provide new potential prognostic, diagnostic, and therapeutic implications for HCC patients.

## Author contributions

YS and HS conceived of and designed the experiments. QX, HS, and YS analyzed the data; HY and ZW prepared the figures and tables; YS, QX, and ZD drafted the manuscript or revised it critically for important content. QX and DZ contributed equally to this work and should be considered co-first authors.

**Conceptualization:** Yifan Sun, Haoming Shen.

**Data curation:** Qingqing Xie, Yifan Sun, Haoming Shen.

**Formal analysis:** Qingqing Xie, Huifeng Ye, Zhitong Wu, Yifan Sun.

**Funding acquisition:** Yifan Sun, Haoming Shen.

**Software:** Huifeng Ye, Zhitong Wu.

**Writing – original draft:** Qingqing Xie, Di Zhang, Yifan Sun.

**Writing – review & editing:** Qingqing Xie, Di Zhang, Yifan Sun.

## Acknowledgments

Thanks Sun&Sunny, Tina, we are indispensable.

## Supplementary Material


