# Supplementary material for: Identification of key snoRNAs serves as biomarkers for hepatocellular carcinoma by bioinformatics methods
Source: Medicine (Baltimore). 2022 Sep 30;101(39):e30813. doi: 10.1097/MD.0000000000030813 (PMC9524901; doi:10.1097/MD.0000000000030813)

Supplementary Figure 1. Flowchart of this study. TGCA, The Cancer Genome Atlas; ICGC, International Cancer Genome Consortium; AUC, Area Under Curve; GSEA, Gene Set Enrichment Analysis; GSVA, Gene Set Variation Analysis; snoRNA, small nucleolar RNA

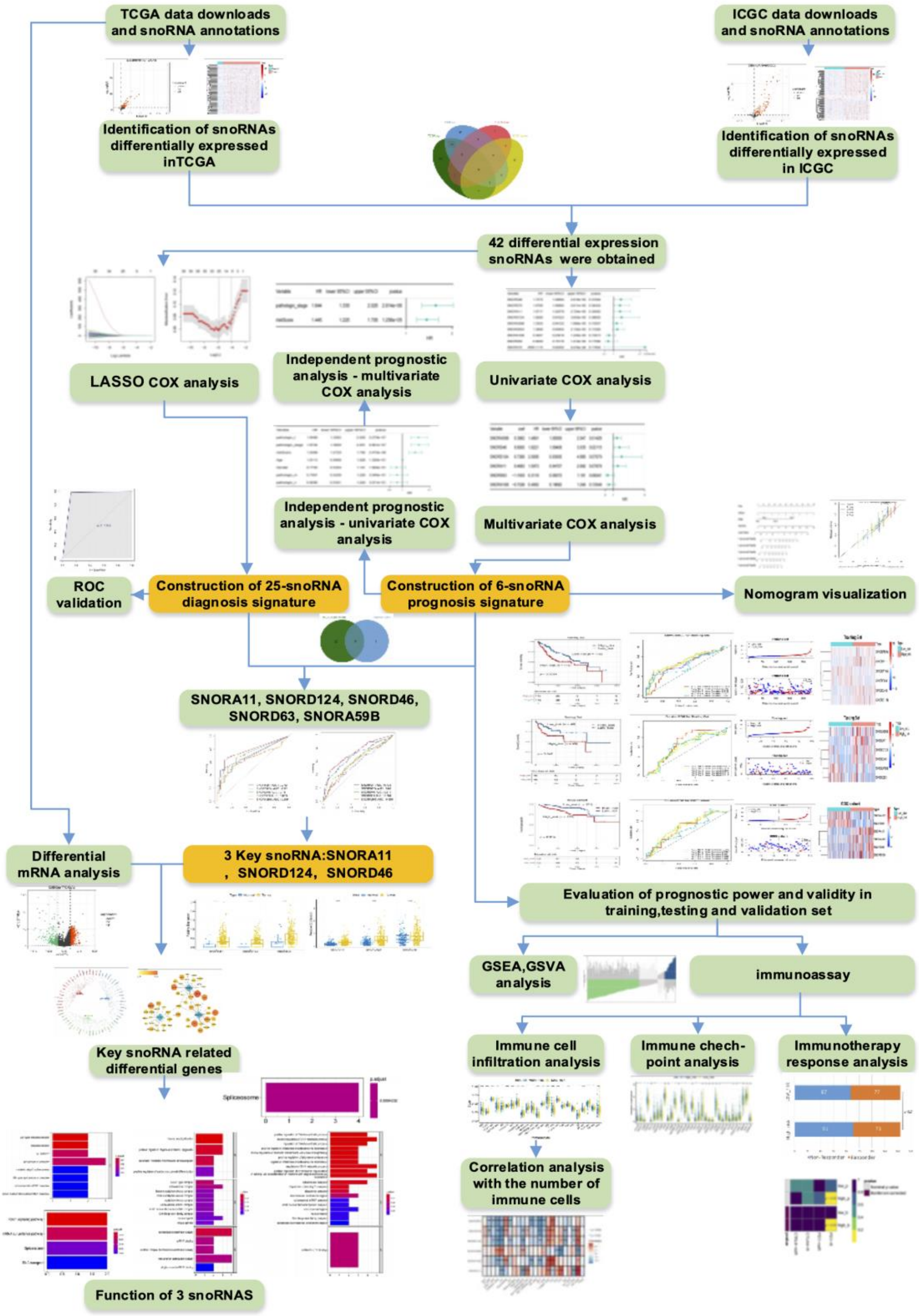

Supplement: Supplementary file 1 [file medi-101-e30813-s001.pdf]
